# Sarcoidosis: Key disease aspects and update on management

**DOI:** 10.1016/j.clinme.2025.100326

**Published:** 2025-05-15

**Authors:** Robina K. Coker, Kathy M. Cullen

**Affiliations:** a Respiratory Medicine, Hammersmith Hospital, Imperial College Healthcare NHS Trust, London W12 0HS, United Kingdom; b Consultant Respiratory Physician, Belfast Trust, Interim Director of Centre for Medical Education, Queens University, Belfast BT7 1NN, United Kingdom

**Keywords:** Antifibrotic, Arrhythmia, Granuloma, Hypercalcaemia, Uveitis

## Abstract

Sarcoidosis is a complex disease of unknown origin, primarily affecting the lungs but capable of involving almost any organ. Diagnosis is challenging due to the lack of specific markers and requires assessment of clinical features, careful imaging, exclusion of other conditions and, ideally, a tissue biopsy demonstrating non-necrotising granulomas.

Over 90% of patients have pulmonary involvement, presenting with symptoms like dry cough and breathlessness, along with systemic signs such as fever and weight loss.

Extrapulmonary manifestations occur in about 30% of cases and can affect any organ, including the heart, nervous system and eyes.

Management involves a multidisciplinary approach with some patients requiring immunosuppressive and antifibrotic therapies. Despite generally good outcomes, sarcoidosis can lead to significant morbidity and mortality, particularly from pulmonary and cardiac complications. Emerging treatments like infliximab and nintedanib offer hope for refractory cases, although they carry risks of serious infections and other side effects.

**Key Points**

 

• Sarcoidosis is often a diagnosis of exclusion, requiring multidisciplinary (MDT) discussion and preferably tissue diagnosis before starting treatment.
• Erythema nodosum does not usually require corticosteroids.
• Sarcoidosis should be considered in new cardiac arrhythmias; early specialist cardiology advice is required with consideration of implantable cardioverter defibrillator (ICD).
• Standard inactive (25 hydroxy-) vitamin D assays do not reliably measure vitamin D status in sarcoidosis.
• Acute anterior uveitis requires emergency ophthalmology assessment to prevent irreversible blindness.

Alt-text: Unlabelled box

## Introduction

**Fig. 1:**
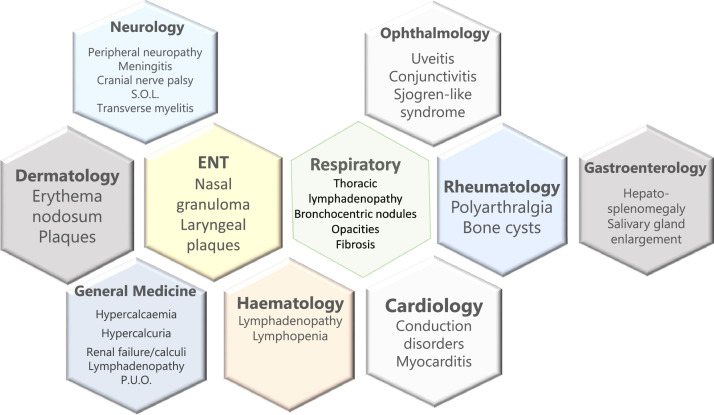
Multisystem manifestations of sarcoidosis. Sarcoidosis can present to any specialty with a range of pulmonary and/or extrapulmonary manifestations.

**Fig. 2:**
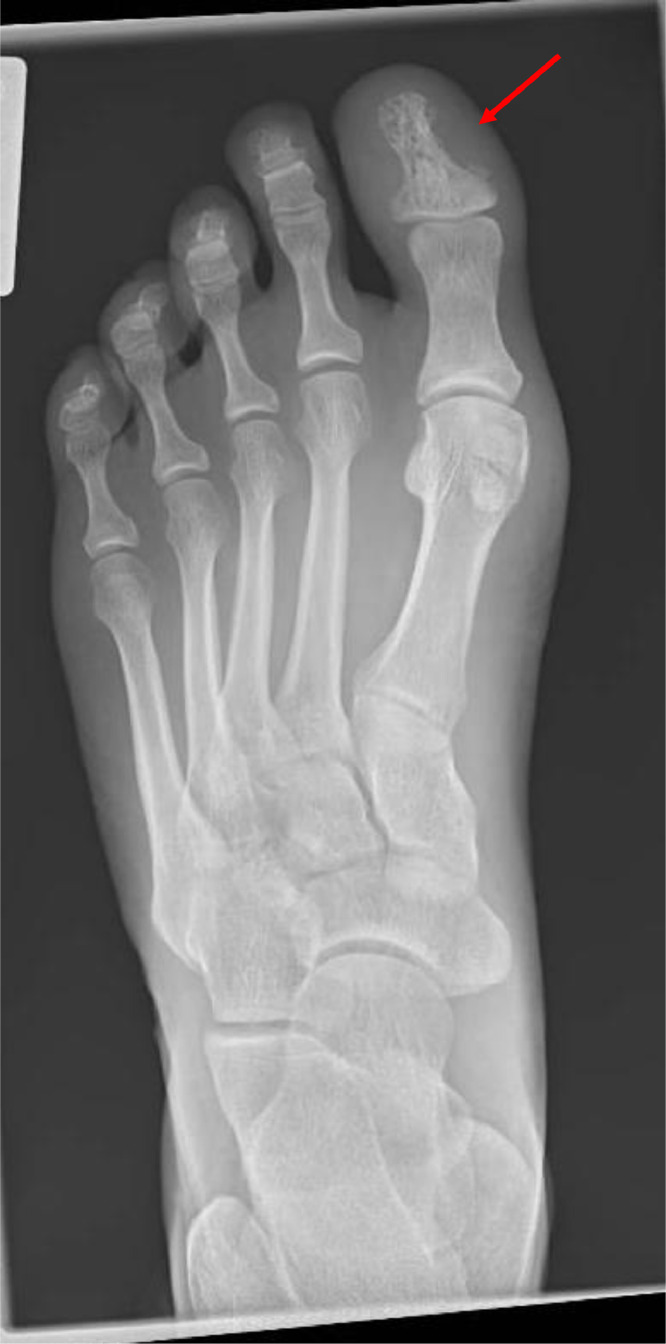
Radiographic appearance of sarcoidosis affecting bone. Typical radiographic appearance of bone involvement by sarcoidosis: lace-like honeycomb appearance of sarcoidosis involving first metatarsal (arrowed) on plain radiograph.

Reported prevalence of sarcoidosis, whose aetiology remains unclear, varies widely, with precise estimates often unavailable owing to phenotypic heterogeneity, diagnostic challenges and variable data quality. Estimates range from 1 per 10,000 (UK) to 6 per 10,000 (USA) and 16 per 10,000 (Sweden). Over 90% of patients have thoracic disease including isolated lymphadenopathy; around 30% have extrapulmonary manifestations^1^ with multiple potential presentations (Fig. 1, Fig. 2). Lymphopenia, hypercalcaemia, raised serum soluble interleukin-2 receptor (sIL-2R), serum or cerebrospinal fluid ACE levels and/or lymphocytosis on bronchoalveolar lavage suggest active disease. Diagnosis requires consistent clinical features; exclusion of other differentials including tuberculosis (TB) and lymphoma; and, preferably, tissue biopsy showing non-necrotising granulomatous inflammation without evidence of TB. Successful management usually requires multidisciplinary collaboration. Pharmacological approaches include corticosteroids, immunosuppression, biologicals and/or antifibrotic therapy. Comorbidities and/or drug toxicities may complicate treatment.

### Respiratory presentations

Most patients have thoracic involvement.^1^ Even conservative European and North American estimates place sarcoidosis as the second commonest long-term pulmonary condition affecting young adults.

Dry cough and exertional breathlessness are common.^1^^,^^2^ Patients may report chest tightness and/or atypical or pleuritic chest pain,^3^ sweats, fevers, malaise and weight loss. Fatigue affects up to 80%.^4^ Respiratory examination, usually normal, does not predict disease extent or severity. Wheeze and stridor are occasional findings in airway involvement. While infrequent, crackles may reflect pulmonary fibrosis. Finger clubbing is rare.^5^

**Fig. 3:**
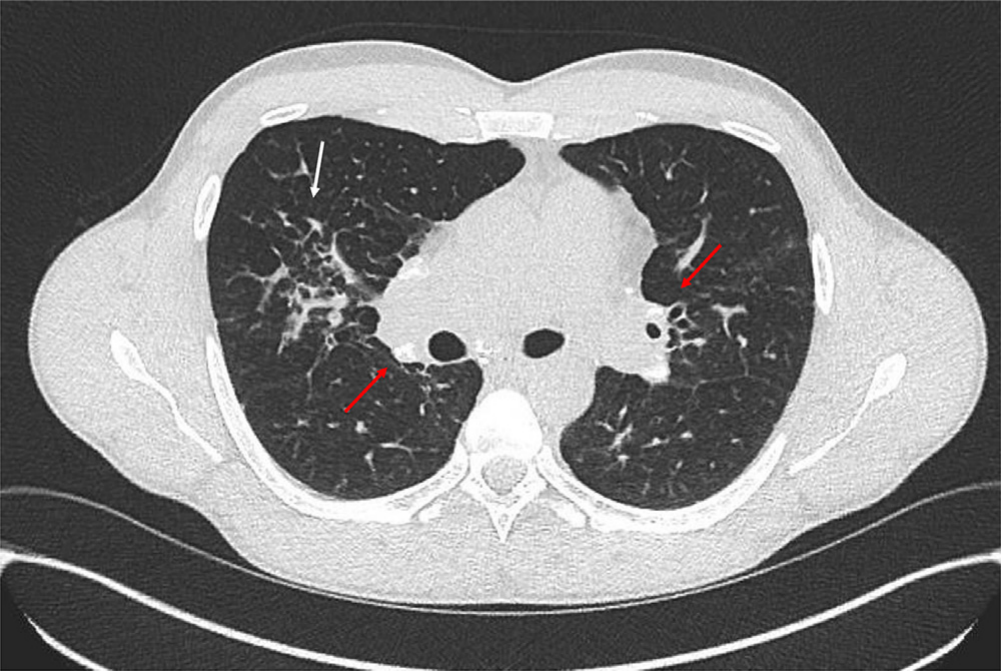
Typical appearances on HRCT. Typical appearances of thoracic sarcoidosis on HRCT: calcified hilar and mediastinal nodes (red arrows), and parenchymal nodularity (white arrow).

Chest radiograph (CXR) and high-resolution computed tomography (HRCT) appearances can be non-specific, mimicking TB, lymphoma, malignancy or interstitial lung disease (ILD). Bronchocentric micronodules (1–3 mm) on HRCT, especially affecting mid and upper zones, are classic features (Fig. 3), conglomeration of nodules around bronchovascular structures causing ‘bronchovascular beading’. Granulomas may coalesce into larger nodules or masses. Micronodule clustering around a larger central nodule, the ‘galaxy sign’, is not pathognomonic.

Diagnostic yield from endobronchial biopsy is 40–60%,^6^ and 40–90% from transbronchial biopsy (TBBx).^7^ Endobronchial ultrasound-guided transbronchial node aspiration (EBUS-TBNA) has transformed diagnosis of nodal disease with yields of 54–93%.^8^ UK recommendations^9^ advise EBUS-TBNA in thoracic lymph node involvement, and TBBx in parenchymal disease. Some centres use transbronchial cryobiopsy in parenchymal disease.

### Classic acute presentations

The acute, self-limiting Löfgren syndrome comprises ankle arthritis, bilateral (BHL) or right hilar lymphadenopathy and erythema nodosum, a panniculitis.^10^ Less common is Heerfordt’s (uveo-parotid syndrome), comprising fever, parotid enlargement, anterior uveitis and facial nerve palsy.

HRCT may be unnecessary where diagnosis is straightforward and the condition mild and self-limiting; but is otherwise nearly always essential. HRCT helps refine the differential diagnosis and guide the choice of bronchoscopy or surgical lung biopsy in the absence of more accessible biopsy targets, including peripheral lymph nodes or skin lesions. Biopsy is not usually required in Löfgren syndrome, or in longstanding pulmonary disease with a typical presentation and stable typical imaging.

### Cardiac presentations

Granulomatous inflammation can cause an infiltrative cardiomyopathy, affecting around 25% of patients with multisystem sarcoidosis and associated with significant morbidity and mortality. Presentations include heart failure, conduction abnormalities, ventricular arrhythmias or sudden cardiac death.^11^ Cardiac sarcoidosis must be considered if patients report palpitations, chest pain or unexplained breathlessness, and in the differential diagnosis of cardiac arrhythmias. ECG, while insensitive and non-specific, is recommended as baseline screening. Symptoms mandate a low threshold for echocardiogram, ambulatory ECG monitoring and cardiac MRI,^12^^,^^13^ followed by FDG PET CT if clinical suspicion remains high. Endomyocardial biopsy has a low diagnostic yield and is now rarely performed. Early specialist cardiology input and consideration of ICD are essential. The possibility of sarcoidosis-associated pulmonary hypertension, whether consequent upon pulmonary fibrosis or a standalone vasculopathy, should also be considered.

### Hypercalcaemia

Sarcoid granulomas can cause parathyroid hormone (PTH)-independent hypercalcaemia through enhanced conversion of 25-hydroxyvitamin D to 1,25-dihydroxyvitamin D. The incidence of hypercalcaemia as the presenting symptom in sarcoidosis is around 3%; hypercalcaemia is reported in around 10% of cases. 1,25-dihydroxy-vitamin D testing is key to diagnosis. Patients with active sarcoidosis may be deficient in 25-hydroxy-vitamin D with normal 1,25-hydroxyvitamin D levels. Vitamin D supplementation based on 25-hydroxy-vitamin D levels can cause hypercalcaemia and acute kidney injury.^14^ If initiated, calcium and/or vitamin D supplementation thus mandates regular serum calcium monitoring.

### Neurological presentations

Around 10% of patients have neurological involvement, which can affect any part and be difficult to diagnose confidently without biopsy confirmation. Facial or optic nerve neuropathies are relatively common, but patients may present with hypothalamic involvement causing diabetes insipidus, pachymeningitis causing seizures and headache, or meningoencephalitis. Steroid monotherapy is not generally successful in meningoencephalitis, and intense therapy regimes are often recommended.^15^

### Uveitis

All patients with eye symptoms require baseline ophthalmic review. A red, painful eye with blurred vision may indicate acute anterior uveitis and mandates urgent ophthalmology assessment. Prompt treatment is required to preserve vision, usually initially with topical steroids. Systemic corticosteroids may be necessary, as may steroid-sparing therapies including methotrexate, azathioprine or mycophenolate mofetil. Biologics, including adalimumab, have been used with success.

### Pharmacological considerations

While outcomes are generally positive, sarcoidosis nevertheless confers increased mortality.^16^ Self-limiting without treatment in around 30%, severe, progressive disease may arise in another 30%, requiring long-term pharmacotherapy.^9^ Pulmonary disease accounts for up to 70% of deaths, cardiac disease for most of the remainder. Fatal comorbidities may complicate steroid treatment or alternative immunosuppression, including hypertension, coronary artery disease, diabetes, osteoporosis and atypical infection.

Corticosteroids, widely used for decades, remain first-line treatment unless contraindicated. Given the risk of treatment-related comorbidities and absence of specific prognostic indications, current UK recommendations only advise starting pharmacological therapy in pulmonary sarcoidosis if there is a potential risk of a fatal outcome, permanent disability or unacceptable loss of quality of life.

The precise starting dose of corticosteroid depends on clinical setting, and the rate of subsequent tapering is determined by individual response. A typical initial treatment regimen in the absence of life-threatening disease is prednisolone 20–40 mg daily for 4–6 weeks, followed by slow tapering to maintenance of 5–10 mg daily. Intravenous methylprednisolone is usually required for cardiac or neurological disease. Attention should be paid to gastric protection, blood pressure, glucose monitoring and early bone density measurement.

Hydroxychloroquine is usually recommended primarily for fatigue, joint or skin disease, but sometimes as a steroid-sparing agent. Other second-line agents include methotrexate, azathioprine, mycophenolate, and leflunomide.

There is no evidence for the efficacy of inhaled steroids, and routine use is not recommended.^17^ Clinical experience suggests that they are sometimes useful for cough and/or airflow obstruction. Asthma is a common comorbidity and should be addressed separately.

Treatment aims to improve symptoms, physiology and imaging, and may necessitate a prolonged maintenance period. Current advice is to attempt to withdraw prednisolone gradually every 6–12 months, with careful monitoring for adverse effects including diabetes, osteoporosis and atypical infection.

Before considering second-line immunosuppression, the diagnosis and treatment adherence should be reviewed. Inability to taper oral prednisolone doses below 10–15 mg daily, progressive pulmonary disease, steroid intolerance, relative contraindications including morbid obesity, diabetes, osteoporosis or hypertension, may all necessitate introduction of alternative agents. Second-line immunosuppression is usually started by a specialist with expertise in sarcoidosis. Methotrexate, azathioprine, mycophenolate and leflunomide all confer substantial risks of toxicity including teratogenicity, myelosuppression, hepatotoxicity and atypical infection. Baseline and regular blood monitoring throughout treatment are essential, as is clinical experience to facilitate prevention and management of toxicities.

Biologicals and antifibrotics are generally only considered when second-line agents have failed; they are usually only available in specialist centres. Infliximab appears to improve disease control^18^ but confers a risk of atypical infection. In 2023, the UK National Institute for Health and Care Excellence (NICE) approved infliximab for refractory sarcoidosis (excluding neurosarcoidosis). Patients should be screened for latent TB infection beforehand, and latent TB treated first. Infliximab is given initially every 2 weeks, then every 4–8 weeks as maintenance.

Nintedanib, an antifibrotic originally licensed for idiopathic pulmonary fibrosis, is now licensed for sarcoidosis-related progressive pulmonary fibrosis (PPF).^19^ Side effects include diarrhoea and hepatotoxicity; continued blood monitoring is required. Early concerns about increased bleeding risk may be allayed by a low incidence (0.29%) in the real-life EMPIRE study.^20^

Fatigue, always challenging, negatively impacts quality of life. Anaemia, iron deficiency, hypercalcaemia, thyroid dysfunction, drug toxicities and sleep disorders should be excluded. Patients should be signposted to sources of support including national charity Sarcoidosis UK: https://www.sarcoidosisuk.org/

Currently recruiting, international multicentre randomised controlled trials raise the prospect of novel, targeted and more effective pharmacological approaches.

## Conclusion

Sarcoidosis is a multisystem disease. Optimum management requires specialist input with multidisciplinary collaboration to enhance communication, reduce hospital visit frequency, and facilitate personalised clinical assessment and management plans.

## Funding

This research did not receive any specific grant from funding agencies in the public, commercial or not-for-profit sectors.

## Declaration of generative AI and AI-assisted technologies in the writing process

During the preparation of this work the author(s) used Copilot as a summary tool to draft the abstract. After using this tool, the authors reviewed and edited the abstract as needed and take full responsibility for its content.

## Consent for publication

Not applicable.

## CRediT authorship contribution statement

**Robina K. Coker:** Writing – original draft. **Kathy M. Cullen:** Writing – review & editing.

## Declaration of competing interest

The authors declare that they have no known competing financial interests or personal relationships that could have appeared to influence the work reported in this paper.
